# The Importance of Right Otitis Media in Childhood Language Disorders

**DOI:** 10.1155/2012/818927

**Published:** 2012-03-26

**Authors:** Paulino Uclés, María Francisca Alonso, Elena Aznar, Carlos Lapresta

**Affiliations:** ^1^Institute Aragones of Health Sciences, Miguel Servet University Hospital, 50009 Saragossa, Spain; ^2^Clinical Neurophysiology Service, Miguel Servet University Hospital, 50009 Saragossa, Spain; ^3^Children Hospital Miguel Servet “ORL Service”, 50009 Saragossa, Spain

## Abstract

Studies relating chronic otitis media and language disorders in children have not reported consistent findings. We carried out the first selective study aimed at discerning the role of chronic right otitis media in children less than 3 years of age in language development. A total of 35 children were studied using a full linguistic protocol, auditory brainstem responses, and middle latency responses. Twelve children had a history of chronic exclusive right otitis media. Seventeen age-matched children were selected as controls. Also, three children having a history of chronic left otitis media were compared with three age-matched controls. Linguistic tests showed significant differences between patients and controls in phonetic, phonological, and syntax scores but not semantics. Correlation studies between linguistic scores and auditory evoked responses in the whole cohort showed a significant coefficient in phonetic and phonological domains. These results emphasize the causative effect of right ear chronic otitis media and indicate that it mainly impairs phonetic and phonological coding of sounds, which may have implications for prophylactic treatment of at-risk children.

## 1. Introduction

In recent decades, neuropsychology has provided consistent data on brain topography for different functions and association areas, and clinical neurophysiology has developed techniques for event-related potentials to accurately measure brain functions. These advancements have expanded the possibilities for investigating both language and audition by joint methods. In clinical practice, neuropaediatrics consulting face an increasing number of language disorders, which sometimes constitute a challenge for diagnosis at the level of impairment; thus, language disorders in children have to be defined by exclusion in most cases [1]. Verbal language is a conventional system of signs by which individuals communicate with each other, and maturation of the nervous system encompasses language development. Acquisition of language starts the first day of extrauterine life, and several language milestones, also called critical periods, have been defined over the maturation period [2]. Myelination of central prethalamic acoustic pathways occurs during the first year of life, whereas much slower myelination of the postthalamic pathway takes five years. The primary auditory cortex shows a peak of synaptic growth during the first two years of life, that is, the plastic changes for hearing are most conspicuous at this age. In 90% of children a functional asymmetry is permanently confined to the left hemisphere by 5 years of age. In right-handed subjects, the auditory association area located at the upper temporal gyrus is greater in size than the same area in the right hemisphere, and the temporal-occipital region of the left hemisphere is larger than the same area in the right, but the reverse occurs with frontoparietal areas. A recent investigation of hearing using fMRI and dichotic listening showed that the functions of both temporal lobes are not identical, as the left temporal lobe specializes in sequential inputs and language processing and the right temporal lobe in melodies, surrounding noise, and prosody [3]. These facts strongly suggest a correlation between anatomical asymmetries and left hemispheric specialization for language, indicating that the auditory pathways are the main channels through which we acquire language. In addition, humans have contralateral, as well as ipsilateral, cortical projections in both the left and right ears. This anatomical distribution allows one to find an acoustic source by a difference of milliseconds in the input reception, with the contralateral radiations being the first to reach the cortex. This perceptual system depends on a series of highly complex mechanisms, and their impairment at any specific level entails more or less serious consequences, depending on which level is affected.

Transient impairment in the middle ear, such as acute otitis media (AOM), and its consequent hearing loss is considered a minor cause of language disorder. The picture that defines AOM is exudate in the cavity derived from inflammation caused by bacteria, which is commonly associated with upper airway infection [4]. AOM may occur as early as the first month of life, but by 3 months 13% of children have suffered a single episode. The chance of experiencing AOM increases with age: 60% at one year, 70% at 3 years, and 80% at 4 years [5]. Starting with a single episode, repeated episodes, mainly in cold seasons, cause the middle ear to be full of fluid, a condition known as otitis media with effusion (OME). This implies a fluctuating conductive loss of hearing in the range of 15–40 dB [6]. Significant hearing loss has been shown in children with a history of OME in the first 3 years of life compared to children who had no otitis [7]. Fluctuations in the hearing threshold during the first 3 years of life make the maturation processes difficult during critical periods when the perceptual attentions for sounds, especially for those of maternal language, are being refined [8].

Over the last three decades, a number of studies have aimed to find relationships between recurrent otitis media in infancy and language development. We have reviewed papers relating both terms in the PubMed and Cochrane databases, limiting the search to randomized controlled studies and meta-analyses. A total of 43 papers were evaluated according to criteria for the method of case selection, basal information, evaluator blindness, and type of measures for hearing and language. Only 12 papers were fit for our purpose. Though a great amount of information was provided, the relationship between OME in infants and language disorders is still controversial [9]. A variety of findings have focused on partial aspects of language. Several studies [10–13] found impaired categorization of speech stimuli in children with an early history of OME. Nelson et al. [14] found impaired phonological consciousness [15] in children with an early incidence of otitis media. Menyuk [8] reported a correlation between prolonged periods of otitis media in children who previously had this condition during the first 3 years of life and articulatory and syntactic impairments. Phonological deprivation further impairs written language. Misperception of some phonemes has been shown to induce a poor semantic database, thereby affecting fluent reading and comprehension [16]. Roberts et al. [17] reported lower scores on receptive and productive language tests in children with an early history of OME compared to children of the same age without such a history. In a follow-up study by these authors in the same cohort, they found that the affected children overcame their language deficit by the age of 6-7 years [18]. Winskel [19] also reported improved language test scores in a cohort of children by the age of 7-8 years. These findings seem to suggest that language deficits disappear by age six, but Zumach et al. argued that reductions in the quality of input for language at critical periods of development have consequences that are difficult to compensate [20, 21]. This finding is a most critical point, mainly because follow-up studies of older children and adolescents are lacking. Remarkably, in the three studies above, no significant correlation was found between prolonged hearing loss and language disorders [22]. Overall, we must say that a general tendency exists for children with an early history of otitis media to develop language disorders, but the causative effect is not yet quantified. The studies did not mention the proportion of cases of bilateral or unilateral otitis, which seems a major factor in the variability of findings. Therefore, discriminating the effects of bilateral, unilateral-right, and unilateral-left OME would offer good insight into this topic.

Selective studies on hearing loss in a single ear have been performed only in rats [23, 24], and evidence shows that plastic changes develop in the cortical and midbrain acoustic areas following temporary occlusion of the right ear. Microelectrode recordings strongly correlated with the auditory evoked potentials, suggesting a study in humans to observe the effects of monaural deprivation of sounds caused by OME in children less than 3 years of age. Strict unilateral OME in children is very unusual, but we attempted to find samples, especially of the right ear because of its putative implication in language disorders; the main purpose of this study was to observe the consequences of right-ear OME suffered early in life on language development. Taking into account that the main input for the development of cortical language areas comes from the contralateral ear, we hypothesised that longstanding right ear hearing deprivation during critical periods of development imposes plastic changes on the cortical acoustic areas, and that these changes can be assessed by auditory evoked potentials. A correlation between hearing and language findings would be evidence that plastic changes have occurred in the corresponding areas of the brain.

## 2. Patients and Methods

An observational study was carried out in a cohort of children aged 3–7 years with a documented history of unilateral right or left OME in the first 3 years of life in order to investigate both hearing and language. These age limits were fixed for the study because formal evaluation of language requires a certain level of collaboration on the part of the subjects, and most of the standard tests for the evaluation of language development are designed for children between 3 and 7 years of age. The questionnaires available for children under 3 years of age are quite subjective and based on the parent's information.

The ethical committee of Aragon approved the project (CP14/2011), and the patients were recruited in the consulting room of the paediatric otolaryngology unit of our hospital. The inclusion criteria were: (a) history of unilateral right or left OME in the first 3 years of life, with duration of more than one year, and annotations in the clinical history of repeated episodes over more than one year were necessary, (b) age between 3–7 years, and (c) written consent of the parents. The exclusion criteria were: (a) documented neurological or psychiatric syndromes, (b) sensory-neural hypoacusia, and (c) social or familiar deprivation of language. These later criteria mean that all children were typically developing in other areas and without a diagnosis of a condition that could affect language development.

A total of 35 children were evaluated using auditory evoked potentials and full language tests. The subjects were distributed into two groups: right OME (*n* = 12) and left OME (*n* = 3). Seventeen normal children of the same age recruited in an elementary school were compared with right OME group, and three normal children were compared with left OME group. At the time when the study was conducted those children that had suffered right or left OME attended the hospital for a follow-up control. They mostly presented chronic sings of otitis media: several had residual exudates into the middle ear cavity and others presented different degrees of tympanic retraction or cicatricial changes in the membrane. All clinical histories had annotations of repeated hearing tests over the first three years of life, consisting in a negative Rinne and a Weber lateralized to the affected ear. In six cases there were added audiometries. These data mean that the patients had had a temporary hearing loss of at least a six-month length. The age of each subject is given in Table 1. The latter three patients and controls belong to the left OME group.

### 2.1. Hearing Tests

**Figure 1:**
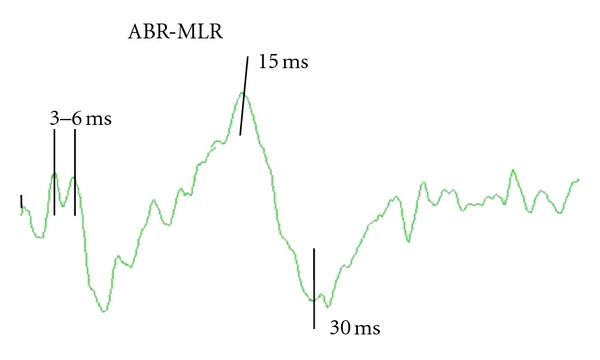
The waveform of ABR and MLR obtained at the same time in a control subject. Time windows for analysis are marked between vertical bars.

The auditory evoked potential was recorded by a cup electrode placed at the vertex (Cz) and reference electrode at the ear lobe (A1, A2). Earphones delivered clicks at 80 dB SPL and a rate of 7 Hz. The right ear was stimulated first, then the left ear, and a masking noise was used at the contralateral ear. With a sample frequency of 547 Hz, 1024 sweeps were averaged with an analysis time of 100 ms, and the waveforms were stored on the hard disk of Medtronic Keypoint (Denmark A/S) equipment for further analysis. Middle latency responses (MLR) provide some components from the subcortical and cortical areas of the acoustic pathway, which are of interest for measuring hearing. By increasing the high-frequency cut-off up to 2 kHz, recording the auditory brainstem response (ABR) and MLR at the same time is possible, so we set the band pass at 4 Hz–2 kHz for simultaneous recording. Taking into account the poor collaborative behaviour of children, this technique is quite convenient [25]. These responses are routinely measured in the time domain, but we were interested in a particular frequency band, the gamma frequency (30–60 Hz), involved in cognitive processes, specifically in the perception of sounds. Consequently, our analysis was performed in the time-frequency domain using a wavelet method, Daubechies wavelet [26]. Changes in power at two selected time windows, a brainstem source between 3–6 milliseconds from the stimulus, onset and a cortical source between 15–30 milliseconds from stimulus onset, were measured. The activity at these two segments provides information on the activity at the brainstem and cortical levels (Figure 1).

### 2.2. Linguistic Tests

Standard neuropsychological tests equivalent to the age of the children in the investigation were used to quantify different domains of language, both in comprehension and production. Phonetic development was assessed by the TAR test [27], which includes a checklist of bisyllabic, trisyllabic, and polysyllabic words intended to provoke a variety of phonemes. The test has been useful for detecting any kind of dyslalia.

Phonological comprehension was assessed by Monfort's test [28]. For language morphology and syntax, we used Aguado's syntax test (TSA) [29]. The TSA is a normalized test providing qualitative variables that can be converted into percentile. Semantic development was assessed by the Peabody Picture Vocabulary Test (PPVT-III) [30]. This test uses pictures to evaluate the vocabulary of children. The results are also transformed into percentile of the normal population for a given age.

### 2.3. Statistical Analysis

The Statistical Package for the Social Sciences (SPSS) was used to analyse our findings. The two-tailed test of significance was used with the significance level set at 0.05. Quantitative variables are provided with central trend indexes (mean, median) and dispersion (standard deviation, range). Qualitative variables are provided as frequency distribution of percentile. Prior to statistical analysis, Kolmogorov-Smirnov and Shapiro-Wilkinson tests were performed to see the adjustment and normal distribution of data. Only the age of patients and controls showed normal distribution. Due to the sample size and nonnormal distribution of data, nonparametric tests for the statistical analysis were used. Bivariant analysis between cases and controls used the Mann-Whitney *U* test, and the correlation between neuropsychological and neurophysiological data was determined by the Spearman rho coefficient.

## 3. Results

Standard tests of language equivalent to the age of the patients and controls provided data on phonetic and phonological development with figures that represent the number of errors, whereas syntax and semantics are represented as percentile of the normal population (Table 2). It has to be pointed out that those higher scores in dyslalia and Monfort's tests mean poor performance (errors) while the reverse is true for syntax TSA and semantic PPVT-III.

The analysis of energy changes in the ABR and MLR time windows was performed using the Daubechies wavelet method. Decomposition of the signal was achieved first, and then the corresponding scale for gamma frequency was found. The total energy at the selective time windows corresponds to the area under a curve in any subject (Table 3).

Frequency values in the descriptive analysis of right OME patients and controls include mean, median and standard deviation, and range (Table 4). Only mean values were used for the comparison between groups using the Mann-Whitney *U* test.  Table 5 shows significant differences in phonetics, phonologic, and syntactic values, and almost significant for semantics, whereas the between-groups age and electrophysiological differences were not significant.

### 3.1. Left Ear Otitis Media

The second group of patients is composed of children with unilateral left ear OME. This group is currently being recruited and we intend to demonstrate the insignificant influence of left OME on language development. Though no statistical analysis is yet possible because of the small size of the sample, these preliminary results emphasize the crucial importance of right ear otitis media in language development, as none of the children with a history of left otitis media in early infancy had a language disorder, despite analysis of hearing on the left acoustic pathway showing lower energy scores than controls (mean ABR controls = 34.26 and mean MLR controls = 45.30; Tables 4 and 6).

### 3.2. Correlation between Linguistic and Electrophysiological Data

Finally, data from linguistic and electrophysiological tests were correlated with right ear otitis media using the Spearman linear regression analysis. Power values for the ABR and MLR time windows were correlated with any particular category of language, and the rho coefficient was calculated with 95% confidence limits.

### 3.3. Auditory Brainstem

**Figure 2:**
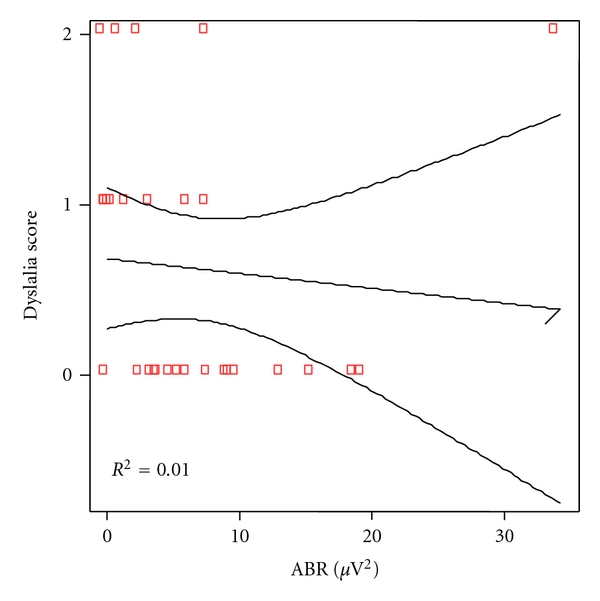
Correlation between phonetic scores and ABR power in patients and controls. Spearman rho coefficient = −0.407 (*P* = 0.028).

**Figure 3:**
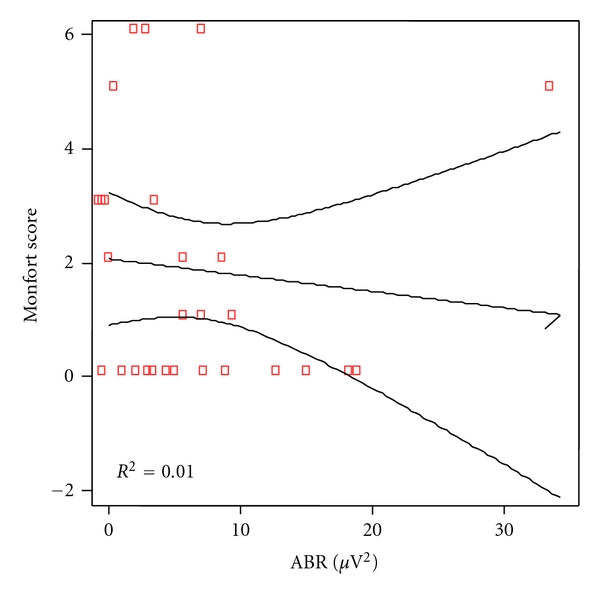
Correlation between phonological scores and ABR power in patients and controls. Spearman rho coefficient = −0.320 (*P* = 0.090).

**Figure 4:**
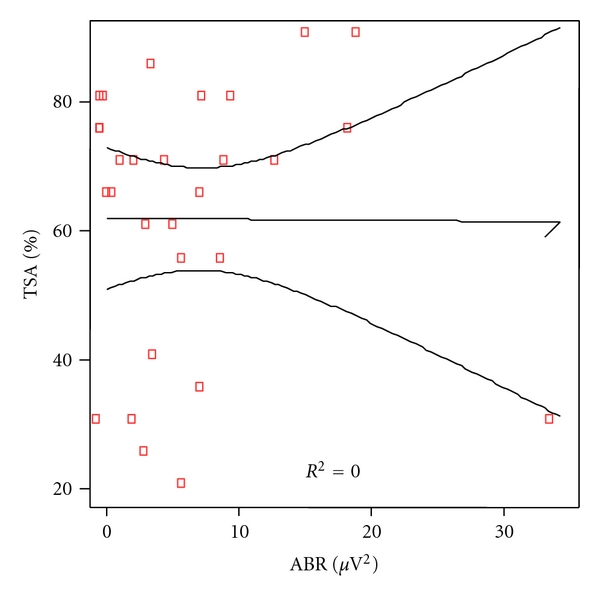
Correlation between morphosyntax scores and ABR power in patients and controls. Spearman rho coefficient = 0.097 (*P* = 0.615).

**Figure 5:**
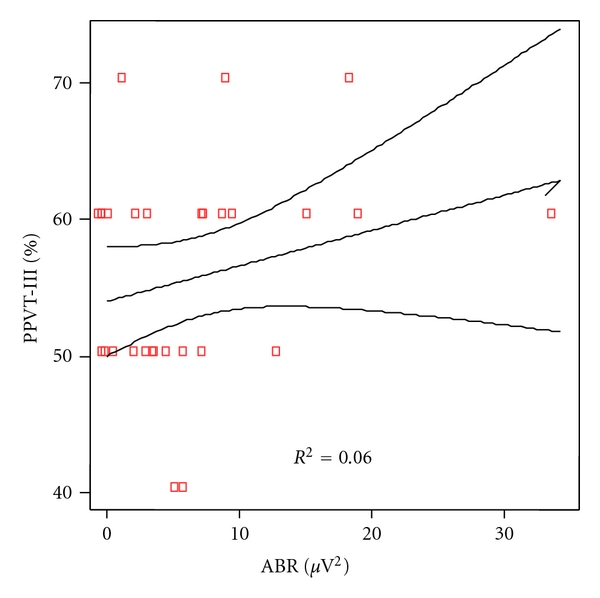
Correlation between semantic scores and ABR power in patients and controls. Spearman rho coefficient = 0.192 (*P* = 0.31).

Analytical correlation for every language levels and power of the ABR was made. Significant correlation was found for phonetic as shown in Figure 2 (*P* = 0.028) but not for phonologic (*P* = 0.090), Figure 3 nor syntax (*P* = 0.615), Figure 4, and semantic (*P* = 0.318), Figure 5.

### 3.4. Auditory Cortex

**Figure 6:**
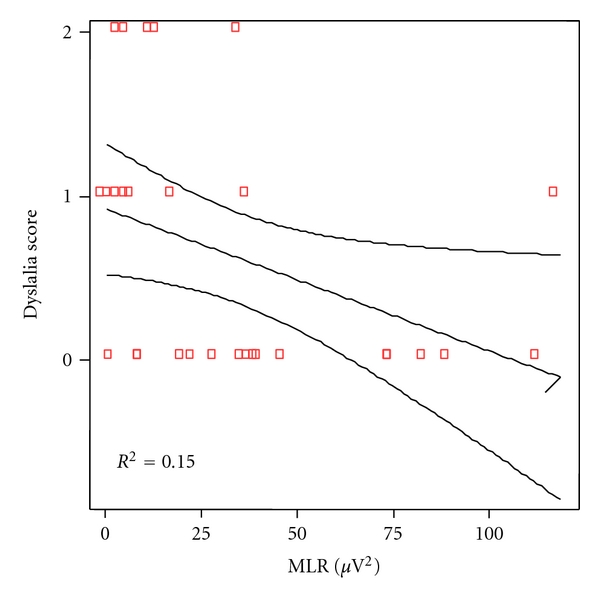
Correlation between phonetic scores and MLR power in patients and controls. Spearman rho coefficient = −0.487 (*P* = 0.007).

**Figure 7:**
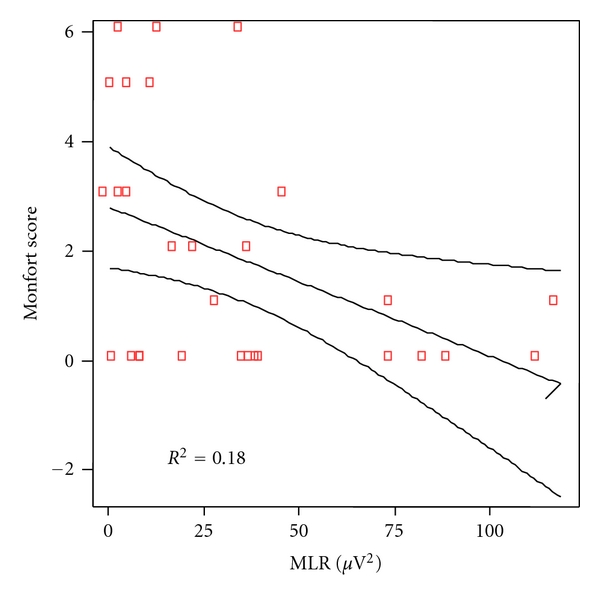
Correlation between phonological scores and MLR power in patients and controls. Spearman rho coefficient = −0.433 (*P* = 0.019).

**Figure 8:**
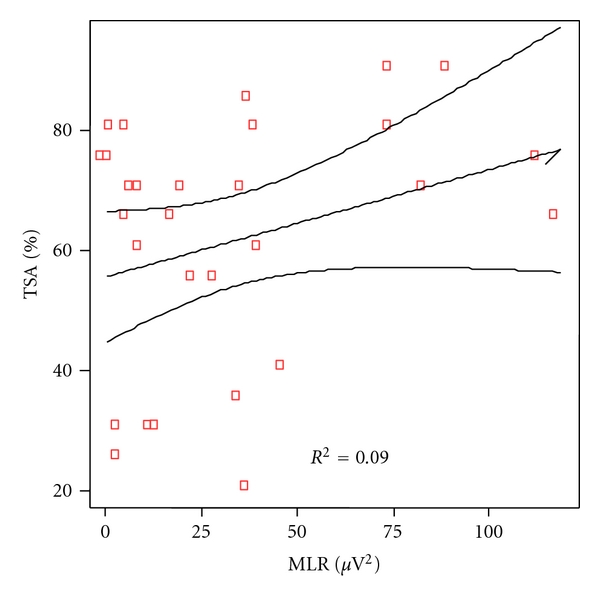
Correlation between morphosyntax and MLR power in patients and controls. Spearman rho coefficient = 0.191 (*P* = 0.322).

**Figure 9:**
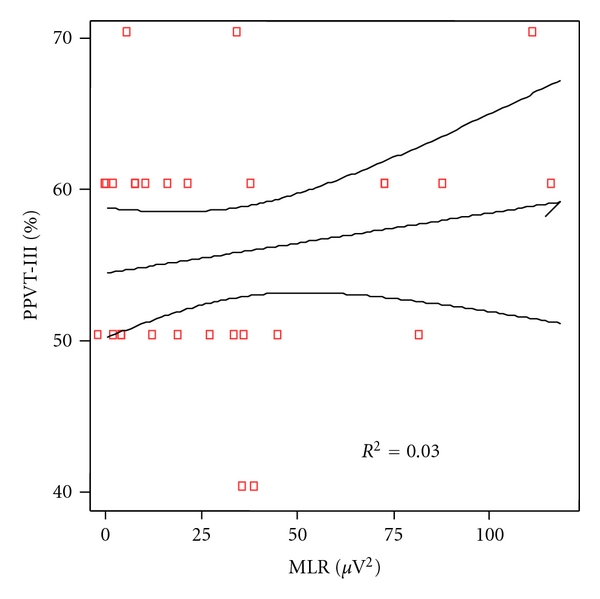
Correlation between semantic scores and MLR power in patients and controls. Spearman rho coefficient = 0.045 (*P* = 0.817).

The same analytical correlation was made between the linguistic scores of any particular category of language and the power of the gamma band of auditory middle latency responses. At cortical level, the correlation coefficient was significant for phonetic (*P* = 0.007), as shown in Figure 6 and for phonologic (*P* = 0.019), Figure 7, but not for syntax (*P* = 0.322), Figure 8, nor semantic (*P* = 0.817), Figure 9.

## 4. Discussion

Specific language disorder in children has a heterogeneous clinical presentation. Sometimes the clinical picture is an impairment of phonological programming, in other cases a phonologic-syntactic disorder, or even verbal auditory agnosia, is present. Our findings in humans are in line with those of Popescu and Polley in animals [23] but differ in an important aspect: the unilateral ear's occlusion that we studied allows the type of linguistic impairment to be determined. The conductive hearing loss associated with childhood ear infections that produce long-lasting deficits in auditory perceptual acuity, much like amblyopia in the visual system, was compared in Popescu and Polley's work to changes produced in infant, juvenile, and adult rats after transient monaural deprivation. In contrast, our method cannot match the in situ findings (distortion of tonotopic maps, weakening of the deprived ear representation with strengthening of the open ear's representation, and disruption of the binaural integration of interaural level differences), but we agree with their results: bidirectional plasticity effects were strictly governed by critical periods, were more strongly expressed in the primary auditory cortex than inferior colliculus, and directly impacted neural coding accuracy, in keeping with greater energy losses in MLR power than ABR power and producing a greater impairment in phonetic-phonological coding than in syntactic-semantic production. In fact, ABR power correlates significantly with phonetic scores while MLR power correlates well with both phonetic and phonologic scores. These facts are in keeping with the contribution of brainstem and cerebral cortex to plasticity changes that take place as the effect of monaural deprivation. In animal experimentation [23] the scope of reorganization is most striking in the cortex and not at the lower parts of the central auditory pathways. It is shown that by combining bilateral recordings in the cortex and the central nucleus of the inferior colliculus with detailed ABR measurements it has been possible to identify reorganizational features that cannot be explained by low-level changes in the auditory system and others that must be. The scope and sensitivity of cortical reorganization in response to experimental manipulation is remarkable given the intimate association between the auditory cortex elementary properties of auditory perception [31]. The electrophysiological mean values of our right OME patients were not significantly different from control values but this could be because of the small sample size. The lack of significant correlation with semantic scores is, in our view, due to the complex cerebral functions involved in semantic; its measurement by short and middle latency responses (ABR-MLR) are not appropriate and long latency evoked potentials should be used for this purpose.

Commonalities exist in relation to the ABR features of the unilateral ligated ear in rats and our ABR-MLR findings in children. In the animal experiments, comparison of representative 80 dB click-evoked ABR waveforms demonstrated that responses from the ligated ear are almost completely restored following ligation removal. Quantitative analysis of waves Ia, I, and II, which are known to be generated by the inner ear hair cells, spiral ganglion cells, and cochlear nucleus globular cells, respectively [32, 33], revealed significant attenuation of the response strength for all three peaks with ligation relative to the open ear. Following ligation removal, the amplitudes of waves Ia and I were immediately restored to equivalence with the open ear as to overlap with sham data points, suggesting that the peripheral hearing loss was completely reversed. Wave II response amplitudes continued to exhibit significant attenuation. These data suggest that the residual high-frequency ABR threshold shift likely stemmed from changes in central auditory neurons, as only wave II failed to recover. Extrapolating these findings to the losses in ABR-MLR power found in children with a history of early right ear OME is possible and are in agreement with the short-term effects on language development. The correlations with phonetic, phonologic, and somewhat with syntactic scores give support to establish a pattern of impairment for those children at risk for language problems that differentiate them from typically developing children. At the age of 3, when children normally collaborate in linguistic testing, it could be stated that more than one phonetic and phonologic error plus a syntactic percentile less than 75 and a semantic percentile less than 60 constitute a pattern to alert for early intervention. The kind of intervention could be sound therapy. Based on the audiograms, musical pieces could be customized for every child at risk in order to restore frequency losses.

This investigation was motivated by the lack of selective studies analysing the role of unilateral chronic otitis media in language development. The strict relationship between chronic unilateral right otitis media and language disorder seemed logical to us because the contralateral auditory pathway is the most efficient in hearing and because of location of language in the left hemisphere in the great majority of people. This selective investigation of right otitis media not only demonstrates its implication in the disorder, but indicates the pattern of impairment, with more distorted coding at phonetic and phonological levels than syntactic and semantic levels. Nonselective investigations reported by several authors have associated phonological and morpho-syntactic impairments with otitis in young children [6, 13, 19, 34], and these observations coincide with our findings, but our selective investigation uncovered the disparity in findings among the general studies, as it points to the right acoustic pathway as a major factor for language disorders. Though it has the inherent limitations of small samples sizes, the reason for that is the extreme difficulty in obtaining participants in the consulting rooms that have exclusive unilateral OME. This implies the use of nonparametric statistical tests and creates the need for replication studies. On the contrary, the linguistic tests that we used were strong since it were standardized for the mother language and adapted to the every one's age.

From a linguistic point of view, the direct causative effect of right OME cannot be stated if we adhere to the current theory of specific language disorder. Genetic, familial, and social deprivation of hearing, among many other factors, have to be considered [35]. The present findings argue against the studies dealing with otitis media and language, as prolonged hearing loss is methodologically considered a dependent variable, but it should be taken as an independent factor, balanced among other factors such as genetic predisposition or familial-social deprivation of language. Thus, for an accurate diagnosis, the proportional role of hearing loss derived from chronic otitis media is important.

## 5. Conclusion

The decomposition of factors leading to childhood language disorder is a breakthrough. To date, this study is the first to analyse unilateral hearing deprivation as a factor for language disorders. However, the extreme difficulty in finding isolated cases of unilateral OME in clinical settings, and consequently obtaining ample cohorts, results in a need for replication studies in order to acquire evidence regarding the importance of right ear otitis media in language disorders in children. The findings imply that clinicians should take measures to prevent language disorders, such as early stimulation in at-risk children, or even the incorporation of some type of sounds in their daily life.

## Figures and Tables

**Table 1:** Cohort ages.

Patient^#^	Age (yr, mo)	Control^#^	Age (yr, mo)
1	5,7	1	5,4
2	5,2	2	3,1
3	5,9	3	5,3
4	5,1	4	3,1
5	6,5	5	6,11
6	3,11	6	5,6
7	4	7	3
8	5,1	8	6,7
9	6,1	9	6
10	4,4	10	6,1
11	4,11	11	6,11
12	3,7	12	5,3
13	5,4	13	3,8
14	3,2	14	4,2
15	5,2	15	3,1
		16	5,0
		17	6,0
		18	5,3
		19	5,6
		20	3,1

^#^Number.

**Table 2:** Results of language tests in the right OME group.

Subject number	Group	Phonetic-phonologic		Morpho-syntactic	Semantic
		Dyslalia	Monfort's	Percent TSA	Percent PPVT-III
1	Case	0	3	40	50
2	Case	0	2	55	60
3	Case	2	5	30	60
4	Case	2	6	35	50
5	Case	2	5	65	50
6	Case	1	2	20	40
7	Case	1	5	75	60
8	Case	0	0	60	40
9	Case	2	3	30	60
10	Case	1	6	25	50
11	Case	2	6	30	50
12	Case	1	2	65	60
1	Control	0	1	80	60
2	Control	1	3	75	50
3	Control	0	0	70	60
4	Control	0	1	55	50
5	Control	0	0	80	60
6	Control	0	0	90	60
7	Control	1	3	80	50
8	Control	0	0	90	60
9	Control	0	0	75	70
10	Control	0	0	80	60
11	Control	0	0	85	50
12	Control	0	0	70	50
13	Control	1	1	65	60
14	Control	0	0	70	50
15	Control	1	0	70	70
16	Control	0	0	70	70
17	Control	0	0	60	60

**Table 3:** Power (*μ*V²) in the ABR and MLR time windows in patients and controls.

Number	Right OME	Age yr, mo	ABR *μ*V²	MLR *μ*V²
	Case/control		(3–6 ms)	(15–30 ms)
1	Case	5,7	4.26	47.40
2	Case	5,2	9.40	23.95
3	Case	5,9	34.27	12.73
4	Case	5,1	7.87	35.90
5	Case	6,5	1.11	6.65
6	Case	3,11	6.34	37.87
7	Case	4,0	0.28	2.10
8	Case	5,1	5.72	41.19
9	Case	6,1	0.00	4.45
10	Case	4,4	3.54	4.54
11	Case	4,11	2.64	14.63
12	Case	7,3	0.76	18.57
1	Control	5,4	10.10	75.33
2	Control	3,10	0.21	0.47
3	Control	5,3	2.84	10.18
4	Control	3,10	6.44	29.73
5	Control	6,11	0.25	2.38
6	Control	5,6	15.80	75.03
7	Control	3,0	0.43	6.55
8	Control	6,7	19.65	90.35
9	Control	6,0	18.98	113.51
10	Control	6,1	7.93	40.11
11	Control	6,11	4.05	38.27
12	Control	5,3	19.65	90.35
13	Control	3,8	7.76	118.39
14	Control	4,2	5.08	21.30
15	Control	3,1	1.72	8.12
16	Control	5,0	9.61	36.7
17	Control	6,0	3.67	9.97

**Table 4:** Neuropsychological and neurophysiological frequency values for right OME and controls.

Right OME	Patients				Controls			
	Mean	SD	Median	Range	Mean	SD	Median	Range
Age yr, mo	4,99	0,89	5,08	2,83	5,13	1,22	5,25	3,92
Phonetic score	1.17	0.83	1.00	2.00	0.24	0.44	0.00	1.00
Phonologic score	3.75	2.01	4.00	6.00	0.53	1.01	0.00	3.00
Morphosyntax	44.17	18.69	37.50	55.00	74.41	9.66	75.00	35.00
Semantic	52.50	7.54	50.00	20.00	58.24	7.28	60.00	20.00
ABR *μ*V²	6.35	9.31	3.91	34.26	7.54	6.33	6.45	19.44
MLR *μ*V²	20.84	16.07	16.61	45.30	44.74	39.92	36.76	117.92

**Table 5:** Comparison of mean values in right OME and controls.

Right OME versus controls	Age	Phonetic	Phonologic	Morphosyntax	Semantic	ABR *μ*V²	MLR *μ*V²
Mann-Whitney *U* test	93.500	39.500	19.000	16.000	65.000	78.000	72.000
*P* value	0.706	0.002*	<0.001*	<0.001*	0.076	0.288	0.184

*Significant.

**Table 6:** Neuropsychological and neurophysiological values in left ear OME patients.

Subjects			Linguistic				Hearing	
Number	Case	Age yr, mo	Phonetic-phonologic		Morphosyntax	Semantic	ABR	MLR
			Dyslalia	Monfort	TSA percent	PPVT-III percent	*μ*V²	*μ*V²
1	Left OME	5,4	0	1	80	60	10.10	75.33
2	Left OME	3,1	1	2	75	50	0.21	0.47
3	Left OME	5,3	0	0	70	60	2.84	10.18

	Mean	4,6	0.3	1	75	56.66	4.39	28.66
